# Supplementary material for: Pyruvate Dehydrogenase Kinase 1 inhibition mediated oxidative phosphorylation enhancement in cartilage promotes osteoarthritis progression
Source: BMC Musculoskelet Disord. 2023 Jul 20;24:597. doi: 10.1186/s12891-023-06585-6 (PMC10357736; doi:10.1186/s12891-023-06585-6)
Supplement: Supplementary file 1 — Additional file 1. [file 12891_2023_6585_MOESM1_ESM.zip › KEGG.Gsea.1653622432464/KEGG_PORPHYRIN_AND_CHLOROPHYLL_METABOLISM.html]

Details for gene set KEGG\_PORPHYRIN\_AND\_CHLOROPHYLL\_METABOLISM[GSEA]

|  || Dataset | 55457\_collapsed\_to\_symbols.cls.cls #OA\_versus\_normal.cls.cls #OA\_versus\_normal\_repos |
| Phenotype | cls.cls#OA\_versus\_normal\_repos |
| Upregulated in class | OA |
| GeneSet | KEGG\_PORPHYRIN\_AND\_CHLOROPHYLL\_METABOLISM |
| Enrichment Score (ES) | 0.47483245 |
| Normalized Enrichment Score (NES) | 1.6697032 |
| Nominal p-value | 0.011210762 |
| FDR q-value | 0.027970746 |
| FWER p-Value | 0.357 |
Table: GSEA Results Summary

  

Fig 1: Enrichment plot: KEGG\_PORPHYRIN\_AND\_CHLOROPHYLL\_METABOLISM      
 Profile of the Running ES Score & Positions of GeneSet Members on the Rank Ordered List

  

| SYMBOL | TITLE | RANK IN GENE LIST | RANK METRIC SCORE | RUNNING ES | CORE ENRICHMENT || 1 | ALAS1 | 5'-aminolevulinate synthase 1 [Source:HGNC Symbol;Acc:HGNC:396] | 288 | 0.757 | 0.0530 | Yes |
| 2 | UROS | uroporphyrinogen III synthase [Source:HGNC Symbol;Acc:HGNC:12592] | 323 | 0.735 | 0.1236 | Yes |
| 3 | FECH | ferrochelatase [Source:HGNC Symbol;Acc:HGNC:3647] | 396 | 0.707 | 0.1885 | Yes |
| 4 | UGT2B4 | UDP glucuronosyltransferase family 2 member B4 [Source:HGNC Symbol;Acc:HGNC:12553] | 566 | 0.634 | 0.2385 | Yes |
| 5 | EPRS1 | glutamyl-prolyl-tRNA synthetase 1 [Source:HGNC Symbol;Acc:HGNC:3418] | 581 | 0.629 | 0.3001 | Yes |
| 6 | GUSB | glucuronidase beta [Source:HGNC Symbol;Acc:HGNC:4696] | 904 | 0.541 | 0.3289 | Yes |
| 7 | UROD | uroporphyrinogen decarboxylase [Source:HGNC Symbol;Acc:HGNC:12591] | 905 | 0.541 | 0.3828 | Yes |
| 8 | BLVRA | biliverdin reductase A [Source:HGNC Symbol;Acc:HGNC:1062] | 942 | 0.529 | 0.4327 | Yes |
| 9 | BLVRB | biliverdin reductase B [Source:HGNC Symbol;Acc:HGNC:1063] | 1050 | 0.507 | 0.4748 | Yes |
| 10 | UGT2B17 | UDP glucuronosyltransferase family 2 member B17 [Source:HGNC Symbol;Acc:HGNC:12547] | 2050 | 0.356 | 0.4327 | No |
| 11 | UGT2A3 | UDP glucuronosyltransferase family 2 member A3 [Source:HGNC Symbol;Acc:HGNC:28528] | 2854 | 0.265 | 0.3967 | No |
| 12 | CPOX | coproporphyrinogen oxidase [Source:HGNC Symbol;Acc:HGNC:2321] | 3110 | 0.239 | 0.4006 | No |
| 13 | UGT1A8 | UDP glucuronosyltransferase family 1 member A8 [Source:HGNC Symbol;Acc:HGNC:12540] | 3301 | 0.221 | 0.4079 | No |
| 14 | ALAD | aminolevulinate dehydratase [Source:HGNC Symbol;Acc:HGNC:395] | 3513 | 0.201 | 0.4115 | No |
| 15 | COX15 | cytochrome c oxidase assembly homolog COX15 [Source:HGNC Symbol;Acc:HGNC:2263] | 3727 | 0.183 | 0.4132 | No |
| 16 | PPOX | protoporphyrinogen oxidase [Source:HGNC Symbol;Acc:HGNC:9280] | 4225 | 0.135 | 0.3880 | No |
| 17 | CP | ceruloplasmin [Source:HGNC Symbol;Acc:HGNC:2295] | 4833 | 0.085 | 0.3493 | No |
| 18 | UGT1A10 | UDP glucuronosyltransferase family 1 member A10 [Source:HGNC Symbol;Acc:HGNC:12531] | 4850 | 0.083 | 0.3563 | No |
| 19 | HCCS | holocytochrome c synthase [Source:HGNC Symbol;Acc:HGNC:4837] | 4988 | 0.074 | 0.3530 | No |
| 20 | COX10 | cytochrome c oxidase assembly factor heme A:farnesyltransferase COX10 [Source:HGNC Symbol;Acc:HGNC:2260] | 6092 | -0.012 | 0.2685 | No |
| 21 | HMBS | hydroxymethylbilane synthase [Source:HGNC Symbol;Acc:HGNC:4982] | 6582 | -0.048 | 0.2353 | No |
| 22 | UGT2B11 | UDP glucuronosyltransferase family 2 member B11 [Source:HGNC Symbol;Acc:HGNC:12545] | 7270 | -0.100 | 0.1920 | No |
| 23 | HMOX2 | heme oxygenase 2 [Source:HGNC Symbol;Acc:HGNC:5014] | 7995 | -0.154 | 0.1511 | No |
| 24 | UGT2A1 | UDP glucuronosyltransferase family 2 member A1 complex locus [Source:HGNC Symbol;Acc:HGNC:12542] | 9451 | -0.269 | 0.0648 | No |
| 25 | FTH1 | ferritin heavy chain 1 [Source:HGNC Symbol;Acc:HGNC:3976] | 10033 | -0.325 | 0.0520 | No |
| 26 | ALAS2 | 5'-aminolevulinate synthase 2 [Source:HGNC Symbol;Acc:HGNC:397] | 10247 | -0.346 | 0.0699 | No |
| 27 | UGT2B15 | UDP glucuronosyltransferase family 2 member B15 [Source:HGNC Symbol;Acc:HGNC:12546] | 12225 | -0.652 | -0.0187 | No |
| 28 | HMOX1 | heme oxygenase 1 [Source:HGNC Symbol;Acc:HGNC:5013] | 12421 | -0.714 | 0.0372 | No |
Table: GSEA details [plain text format]

  

Fig 2: KEGG\_PORPHYRIN\_AND\_CHLOROPHYLL\_METABOLISM      
 Blue-Pink O' Gram in the Space of the Analyzed GeneSet

  

Fig 3: KEGG\_PORPHYRIN\_AND\_CHLOROPHYLL\_METABOLISM: Random ES distribution      
 Gene set null distribution of ES for **KEGG\_PORPHYRIN\_AND\_CHLOROPHYLL\_METABOLISM**
